# Supplementary material for: Comparative genomics provides new insights into the diversity, physiology, and sexuality of the only industrially exploited tremellomycete: Phaffia rhodozyma
Source: BMC Genomics. 2016 Nov 9;17:901. doi: 10.1186/s12864-016-3244-7 (PMC5103461; doi:10.1186/s12864-016-3244-7)
Supplement: Additional file 6: — List of orphan genes with links to PFAM (related to Additional file 1: Table S1). (ZIP 1428 kb) [file 12864_2016_3244_MOESM6_ESM.zip › BLAST_HTML_FTR/G05688_P.html]

BLAST Search Results


```
BLASTP 2.2.27+


Reference:
Stephen F. Altschul, Thomas L. Madden, Alejandro A. Schäffer,
Jinghui Zhang, Zheng Zhang, Webb Miller, and David J. Lipman (1997),
"Gapped BLAST and PSI-BLAST: a new generation of protein database
search programs", Nucleic Acids Res. 25:3389-3402.


Reference for
composition-based statistics:
Alejandro A. Schäffer, L. Aravind, Thomas L. Madden, Sergei
Shavirin, John L. Spouge, Yuri I. Wolf, Eugene V. Koonin, and
Stephen F. Altschul (2001), "Improving the accuracy of PSI-BLAST
protein database searches with composition-based statistics and
other refinements", Nucleic Acids Res. 29:2994-3005.


Database: nr
           71,551,133 sequences; 26,053,659,533 total letters


Query= G05688_P

Length=570
                                                                      Score     E
Sequences producing significant alignments:                          (Bits)  Value

emb|CED85496.1|  hypothetical protein [Xanthophyllomyces dendrorh...  1159    0.0  
ref|WP_046785329.1|  c-type cytochrome biogenesis protein CcmI [P...  40.8    3.6  
ref|WP_021785212.1|  MULTISPECIES: c-type cytochrome biogenesis p...  40.8    4.0  
gb|KGO48034.1|  hypothetical protein PEXP_038980 [Penicillium exp...  40.0    8.0  
gb|KGO59786.1|  hypothetical protein PEX2_053640 [Penicillium exp...  40.0    8.6  


 >emb|CED85496.1| hypothetical protein [Xanthophyllomyces dendrorhous]
Length=569

 Score = 1159 bits (2999),  Expect = 0.0, Method: Compositional matrix adjust.
 Identities = 569/569 (100%), Positives = 569/569 (100%), Gaps = 0/569 (0%)

Query  1    MSNCSIRYFISSSSPLSSHGPSEEIPVSKWALVPASPSPSASKDALILLTLFQIPTALLT  60
            MSNCSIRYFISSSSPLSSHGPSEEIPVSKWALVPASPSPSASKDALILLTLFQIPTALLT
Sbjct  1    MSNCSIRYFISSSSPLSSHGPSEEIPVSKWALVPASPSPSASKDALILLTLFQIPTALLT  60

Query  61   FGLTAGSFYMFTRSKGARGLTSAATTSAQQAVARSGESPRQILLKRLDRLESLSNENQQW  120
            FGLTAGSFYMFTRSKGARGLTSAATTSAQQAVARSGESPRQILLKRLDRLESLSNENQQW
Sbjct  61   FGLTAGSFYMFTRSKGARGLTSAATTSAQQAVARSGESPRQILLKRLDRLESLSNENQQW  120

Query  121  SRQLEGRLEKNVALGVESIRSDLSQIRRLLMKDNDRLQSSLSRIERLLEQLPQSSTANQM  180
            SRQLEGRLEKNVALGVESIRSDLSQIRRLLMKDNDRLQSSLSRIERLLEQLPQSSTANQM
Sbjct  121  SRQLEGRLEKNVALGVESIRSDLSQIRRLLMKDNDRLQSSLSRIERLLEQLPQSSTANQM  180

Query  181  TKISSDDSSPPSGGLTAPTQKLVSKPVTSKESIWEAARDSSPFLYGQHPSGGPAKSPAGP  240
            TKISSDDSSPPSGGLTAPTQKLVSKPVTSKESIWEAARDSSPFLYGQHPSGGPAKSPAGP
Sbjct  181  TKISSDDSSPPSGGLTAPTQKLVSKPVTSKESIWEAARDSSPFLYGQHPSGGPAKSPAGP  240

Query  241  ILTDQSPKEEDTPLKYTSAFRPTVQSSDKTPDKADIARWSSLFNKKKQEQNVKGVGFESE  300
            ILTDQSPKEEDTPLKYTSAFRPTVQSSDKTPDKADIARWSSLFNKKKQEQNVKGVGFESE
Sbjct  241  ILTDQSPKEEDTPLKYTSAFRPTVQSSDKTPDKADIARWSSLFNKKKQEQNVKGVGFESE  300

Query  301  IKEARRKAEEEEDRVAQEEEDWKAQKRGWTEDLKPTQKSFVLVNTDEPSNTVVSSSPIEV  360
            IKEARRKAEEEEDRVAQEEEDWKAQKRGWTEDLKPTQKSFVLVNTDEPSNTVVSSSPIEV
Sbjct  301  IKEARRKAEEEEDRVAQEEEDWKAQKRGWTEDLKPTQKSFVLVNTDEPSNTVVSSSPIEV  360

Query  361  NSDHPASSSTRTDDIHHDVLPKGIVEETRPGESKETPVTETAQPEPHVASEADPLLVHQN  420
            NSDHPASSSTRTDDIHHDVLPKGIVEETRPGESKETPVTETAQPEPHVASEADPLLVHQN
Sbjct  361  NSDHPASSSTRTDDIHHDVLPKGIVEETRPGESKETPVTETAQPEPHVASEADPLLVHQN  420

Query  421  NDRQFRSLDRSSTSQTSWSQTPRSGSSTDSSSPDDASTAARSQLKPEKAVNTDTTPSTPS  480
            NDRQFRSLDRSSTSQTSWSQTPRSGSSTDSSSPDDASTAARSQLKPEKAVNTDTTPSTPS
Sbjct  421  NDRQFRSLDRSSTSQTSWSQTPRSGSSTDSSSPDDASTAARSQLKPEKAVNTDTTPSTPS  480

Query  481  ITRPCFPFGFQPHSMASAFAFHFETPRAFRYGFTHPFTRAEAAVKEKENDKAREHEGLPL  540
            ITRPCFPFGFQPHSMASAFAFHFETPRAFRYGFTHPFTRAEAAVKEKENDKAREHEGLPL
Sbjct  481  ITRPCFPFGFQPHSMASAFAFHFETPRAFRYGFTHPFTRAEAAVKEKENDKAREHEGLPL  540

Query  541  GEIKTHGQGCSYWTIINGVRVKRVSSWRR  569
            GEIKTHGQGCSYWTIINGVRVKRVSSWRR
Sbjct  541  GEIKTHGQGCSYWTIINGVRVKRVSSWRR  569


>ref|WP_046785329.1| c-type cytochrome biogenesis protein CcmI [Pseudomonas putida]
Length=396

 Score = 40.8 bits (94),  Expect = 3.6, Method: Compositional matrix adjust.
 Identities = 50/215 (23%), Positives = 95/215 (44%), Gaps = 22/215 (10%)

Query  64   TAGSFYMFTRSKGARGLTSAATTSAQQAVARSGESPRQILLKRLDRLESLSNENQQWSRQ  123
            +A   Y   R+  A    + A  + ++AVA +G  P   LL +  +    +  N+QWS Q
Sbjct  149  SAEGLYFLGRAYMAEQRPADAAKAYERAVALAGRQPE--LLGQWAQALYFAA-NKQWSAQ  205

Query  124  LEGRLEKNVALGVESIRSDLSQIRRLLMK-----DNDRLQSSLSRIERLLEQLPQSSTAN  178
            ++       AL  E++++D +++  L ++     + +R Q ++    RLL QLP+   + 
Sbjct  206  VQ-------ALTDEALKADPNEVTSLGLRGIAAFEGERYQEAIDYWNRLLAQLPEGDASR  258

Query  179  QMTKISSDDSSPPSGGLTAPTQKLVSKPVTSKESIWEAAR-----DSSPFLYGQHPSGGP  233
               +   D ++   GG T   +  V+  +  +  +  A +     D + F++ +  SG P
Sbjct  259  AALQGGIDRAAERLGGNTGKPETPVAARLKVRVELAPALKDKVKPDDTVFIFARATSGPP  318

Query  234  AKSPAGPILTDQSPKEEDTPLKYTSAFRPTVQSSD  268
                A  +   Q P E +  L    A  P ++ SD
Sbjct  319  MPLAAKRVTVAQLPLEVE--LSDADAMMPQMKLSD  351


>ref|WP_021785212.1| MULTISPECIES: c-type cytochrome biogenesis protein CcmI [Pseudomonas 
putida group]
 gb|ERK99007.1| hypothetical protein O999_14250 [Pseudomonas putida LF54]
Length=398

 Score = 40.8 bits (94),  Expect = 4.0, Method: Compositional matrix adjust.
 Identities = 49/200 (25%), Positives = 92/200 (46%), Gaps = 26/200 (13%)

Query  64   TAGSFYMFTRSKGARGLTSAATTSAQQAVARSGESPRQILLKRLDRLESLSNENQQWSRQ  123
            +A + Y   R+  A    + A  + ++AVA +G  P   LL +  +    +  N+QWS Q
Sbjct  149  SAEAMYFLGRAYMAEQRPADAARTFERAVALAGRQPE--LLGQWAQALYFAA-NKQWSPQ  205

Query  124  LEGRLEKNVALGVESIRSDLSQIRRLLMK-----DNDRLQSSLSRIERLLEQLPQSSTAN  178
            L+       AL  E++++D +++  L ++     + +R Q ++   +RLL QLP+  T+ 
Sbjct  206  LQ-------ALTDEALKADPNEVTSLGLRGIAAFEGERYQEAIDYWKRLLAQLPEGDTSR  258

Query  179  QMTKISSDDSSPPSGGLTAPTQKLVSKPVTSKESI----WEAARDS-----SPFLYGQHP  229
               +   D ++   GG  AP Q     PV ++  +     +A +D      + F++ +  
Sbjct  259  AALQGGIDRAAERLGG--APAQATEPAPVAARLKVRVELADALKDKVKPDDTVFIFARAS  316

Query  230  SGGPAKSPAGPILTDQSPKE  249
            +G P    A  +   Q P E
Sbjct  317  NGPPMPLAAKRVTVAQLPIE  336


>gb|KGO48034.1| hypothetical protein PEXP_038980 [Penicillium expansum]
 gb|KGO72042.1| hypothetical protein PEX1_023780 [Penicillium expansum]
Length=1414

 Score = 40.0 bits (92),  Expect = 8.0, Method: Compositional matrix adjust.
 Identities = 64/242 (26%), Positives = 90/242 (37%), Gaps = 37/242 (15%)

Query  191   PSGGLTAPTQKLVSKPVTSKESIWEAARDSSPFLYGQHPSGGPAKSPAGPILTDQSPKEE  250
             PS    +P   L + P        +AA  ++    G   SGG A S A P    Q PK +
Sbjct  877   PSPPTASPVATLAAPPAVGLPKFGDAATSTTTSAIGAPKSGGAATSTAAPAF--QIPKFD  934

Query  251   DTPLKYTSAFRPTVQSSDKTPDKADIARWSSLFNKKKQEQNVKGVGFESEIK--------  302
                   TS   P  Q      D A  +   S F   K      GV F  + K        
Sbjct  935   GA---ATSTAAPAFQIPKF--DGAATSNAPSAFQMPKLSGQATGVNFGDQFKSQSDKTLA  989

Query  303   --EARRKAEE----EEDRVAQEEEDWKAQKRGWTEDLKPTQKSFVLV----------NTD  346
               +A RKA E    EED    E +D + Q+    E     +K  V V          NT+
Sbjct  990   DAKAMRKAAEFDSDEEDEAEWERKDEERQRAKRAEIEAAAKKRAVFVPGVGFKFADDNTE  1049

Query  347   EPSNTVVSSSPIEVNSDHPASSSTRTDDIHHDVLPKGIVEETRPGESKETPVTETAQPEP  406
             EP+ +   ++P++   D  A + T T DI    +       TRP     TP   +++P+P
Sbjct  1050  EPATSAAPATPLQ---DDSAGTITPTPDIFPPFVTPSTAASTRP---NGTPSVSSSKPQP  1103

Query  407   HV  408
               
Sbjct  1104  RF  1105


>gb|KGO59786.1| hypothetical protein PEX2_053640 [Penicillium expansum]
Length=1414

 Score = 40.0 bits (92),  Expect = 8.6, Method: Compositional matrix adjust.
 Identities = 64/242 (26%), Positives = 90/242 (37%), Gaps = 37/242 (15%)

Query  191   PSGGLTAPTQKLVSKPVTSKESIWEAARDSSPFLYGQHPSGGPAKSPAGPILTDQSPKEE  250
             PS    +P   L + P        +AA  ++    G   SGG A S A P    Q PK +
Sbjct  877   PSPPTASPVATLAAPPAVGLPKFGDAATSTTTSAIGAPKSGGAATSTAAPAF--QIPKFD  934

Query  251   DTPLKYTSAFRPTVQSSDKTPDKADIARWSSLFNKKKQEQNVKGVGFESEIK--------  302
                   TS   P  Q      D A  +   S F   K      GV F  + K        
Sbjct  935   GA---ATSTAAPAFQIPKF--DGAATSNAPSAFQMPKLSGQATGVNFGDQFKSQSDKTLA  989

Query  303   --EARRKAEE----EEDRVAQEEEDWKAQKRGWTEDLKPTQKSFVLV----------NTD  346
               +A RKA E    EED    E +D + Q+    E     +K  V V          NT+
Sbjct  990   DAKAMRKAAEFDSDEEDEAEWERKDEERQRAKRAEIEAAAKKRAVFVPGVGFKFADDNTE  1049

Query  347   EPSNTVVSSSPIEVNSDHPASSSTRTDDIHHDVLPKGIVEETRPGESKETPVTETAQPEP  406
             EP+ +   ++P++   D  A + T T DI    +       TRP     TP   +++P+P
Sbjct  1050  EPATSAAPATPLQ---DDSAGTITPTPDIFPPFVTPSTAASTRP---NGTPSVSSSKPQP  1103

Query  407   HV  408
               
Sbjct  1104  RF  1105


Lambda      K        H        a         alpha
   0.310    0.125    0.357    0.792     4.96 

Gapped
Lambda      K        H        a         alpha    sigma
   0.267   0.0410    0.140     1.90     42.6     43.6 

Effective search space used: 6165156672990


  Database: nr
    Posted date:  Sep 23, 2015 12:05 AM
  Number of letters in database: 26,053,659,533
  Number of sequences in database:  71,551,133


Matrix: BLOSUM62
Gap Penalties: Existence: 11, Extension: 1
Neighboring words threshold: 11
Window for multiple hits: 40
```
